# Capturing and Documenting the Wider Health Impacts of the COVID-19 Pandemic Through the Remember Rebuild Saskatchewan Initiative: Protocol for a Mixed Methods Interdisciplinary Project

**DOI:** 10.2196/46643

**Published:** 2023-06-06

**Authors:** Nazeem Muhajarine, James Dixon, Erika Dyck, Jim Clifford, Patrick Chassé, Suvadra Datta Gupta, Colleen Christopherson-Cote, Remember Rebuild Saskatchewan Team

**Affiliations:** 1 Department of Community Health and Epidemiology Saskatchewan Population Health and Evaluation Research Unit University of Saskatchewan Saskatoon, SK Canada; 2 Saskatchewan Population Health and Evaluation Research Unit University of Saskatchewan Saskatoon, SK Canada; 3 Department of History College of Arts and Science University of Saskatchewan Saskatoon, SK Canada; 4 Saskatoon Poverty Reduction Partnership Saskatoon, SK Canada; 5 See Acknowledgements

**Keywords:** COVID-19, Saskatchewan, Canada, mixed methods, interdisciplinary, mental health and substance use, food insecurity, housing precarity, archive

## Abstract

**Background:**

In the Canadian province of Saskatchewan, the global COVID-19 pandemic appeared amidst existing social health challenges in food insecurity, housing precarity and homelessness, poor mental health, and substance misuse. These chronic features intersected with the pandemic, producing a moment in time when the urgency of COVID-19 brought attention to underlying shortcomings in public health services.

**Objective:**

The objectives of the program of research are (1) to identify and measure relationships between the pandemic and wider health and social impacts, namely, food insecurity, housing precarity and homelessness, and mental health and substance use in Saskatchewan, and (2) to create an oral history of the pandemic in Saskatchewan in an accessible digital public archive.

**Methods:**

We are using a mixed methods approach to identify the impacts of the pandemic on specific equity-seeking groups and areas of social health concern by developing cross-sectional population-based surveys and producing results based on statistical analysis. We augmented the quantitative analysis by conducting qualitative interviews and oral histories to generate more granular details of people’s experiences of the pandemic. We are focusing on frontline workers, other service providers, and individuals within equity-seeking groups. We are capturing digital evidence and social media posts; we are collecting and organizing key threads using a free open-source research tool, Zotero, to trace the digital evidence of the pandemic in Saskatchewan. This study is approved by the Research Ethics Board at the University of Saskatchewan (Beh-1945).

**Results:**

Funding for this program of research was received in March and April 2022. Survey data were collected between July and November 2022. The collection of oral histories began in June 2022 and concluded in March 2023. In total, 30 oral histories have been collected at the time of this writing. Qualitative interviews began in April 2022 and will continue until March 2024. Survey analysis began in January 2023, and results are expected to be published in mid-2023. All data and stories collected in this work are archived for preservation and freely accessible on the Remember Rebuild Saskatchewan project’s website. We will share results in academic journals and conferences, town halls and community gatherings, social and digital media reports, and through collaborative exhibitions with public library systems.

**Conclusions:**

The pandemic’s ephemeral nature poses a risk of us “forgetting” this moment and the attendant social inequities. These challenges inspired a novel fusion among health researchers, historians, librarians, and service providers in the creation of the Remember Rebuild Saskatchewan project, which focuses on preserving the legacy of the pandemic and capturing data to support an equitable recovery in Saskatchewan.

**International Registered Report Identifier (IRRID):**

DERR1-10.2196/46643

## Introduction

### Background

On March 11, 2020, the World Health Organization declared a global pandemic caused by a new virus, SARS-CoV-2. Due to the uncertainties about the virulence of COVID-19, the disease caused by SARS-CoV-2, and the need to mitigate the expected demand on the health care system, the federal and provincial governments of Canada imposed strict public health restrictions that limited population movement. These new policies helped to slow the spread of COVID-19, but they also had significant social consequences, such as the pivot to remote work and learning and the closure of many small businesses.

In Saskatchewan, a province in central Canada with a population of just under 1.2 million people, the provincial government introduced public health measures with the intention of dampening the initial wave of the pandemic. The transmission of the virus in Saskatchewan was slow during the first 6 months of the pandemic, and the province reported far fewer cases and deaths due to COVID-19 (58.75 and 0.85 per 100,000) than the Canadian average (180.42 cases and 15 per 100,000 deaths) in that time [1]. Like other regions in Canada, Saskatchewan experienced an economic downturn during the first 6 months of the pandemic, losing 5% of its gross domestic product and recording a 5% increase in unemployment [2].

### COVID-19 in Saskatchewan

By 2021, COVID-19 cases and deaths in Saskatchewan were trending upward, surpassing the Canadian average in January and remaining above the national rates throughout 2022 [1]. In November 2022, Saskatchewan reported the third-highest death rate among Canadian provinces, with 142 deaths per 100,000, exceeding the national average of 123 deaths per 100,000. Despite high rates of transmission and deaths per capita, the Government of Saskatchewan progressively eliminated public health restrictions, arguing that these protocols hindered economic growth and personal freedom [3]. Subsequently, Saskatchewan became one of the first Canadian provinces to relax all pandemic-related restrictions, first doing so in July 2021 and then again comprehensively easing restrictions during the Omicron wave in February 2022 [3,4].

The 2022 policy reversals coincided with a reduction in pandemic-related data collection and reporting, resulting in limited access to publicly available data and the capacity for the public to understand and interpret the ongoing effects of the pandemic [5].^ ^Beyond prioritizing economic issues over public health, the decision to stop sharing COVID-19 data publicly made the province of Saskatchewan stand apart from other provinces and territories. This, in part, made it an ideal case study for further examination. This shift in priorities during the pandemic also exacerbated preexisting public health needs, namely food insecurity, housing precarity and homelessness, mental health, and substance use.

Since March 2020, the urban centers of Regina and Saskatoon have experienced an increased surge in social service usage [6-8], homelessness, and housing precarity [9-11]. Saskatchewan residents also reported higher rates of food insecurity [12], mental distress, and illness [13-16], and the province experienced a drastic increase in substance use-related deaths [17]. Moreover, the government’s decision to stop releasing health data in 2022 led to only a partial understanding of how COVID-19 influenced wider health trends across Saskatchewan.

### Remember Rebuild Saskatchewan: A COVID-19 Project

This protocol paper presents our innovative mixed method protocol for a program of research entitled “Remember Rebuild Saskatchewan*.*” This, in turn, consists of 2 constituent federally funded projects: “Build Back Better: Data and equity needed to drive postpandemic recovery in Saskatchewan,” funded by Canadian Institutes of Health Research (operating grant 478277), and “Saskatchewan’s COVID-19 public archive,” funded by Social Sciences and Humanities Research Council of Canada Partnership Development (grant 356339). The objective of the program of research is to identify and measure relationships between the pandemic and wider health and social impacts, namely, food insecurity, housing precarity and homelessness, and mental health and substance use in Saskatchewan. We also aim to identify how the pandemic affected service providers and service users across the province as they navigated (or attempted to navigate) the COVID-19 pandemic. The overall goal is to preserve data and stories in a digital archive and use them, alongside social media and web archives, to create a social history of the COVID-19 response in Saskatchewan, Canada. The findings from this multidisciplinary and community-focused research model will be used to develop policy, programming, and practice recommendations for policy makers and community-based human service agencies.

## Methods

### Study Setting and Design

This program of study is set in the province of Saskatchewan, Canada. Our interdisciplinary team blends the expertise of many disciplinary experts, for example, epidemiologists, population health researchers, digital humanists, and medical historians, with the expertise and lived experience of community-based partners to create knowledge and build and share a record of the implications of COVID-19 for the province of Saskatchewan. We use a mixed methods study design, including quantitative survey data collection, qualitative engagements with service providers and individuals from equity-seeking groups through interviews and oral histories, as well as social and digital media record collection and analyses. Our interdisciplinary and mixed methods approach allows us to use diverse and complementary techniques for data collection and analysis and to illuminate the impacts of the pandemic in a multifaceted and evidence-informed manner.

### Patient and Public Involvement

The Remember Rebuild Saskatchewan project is heavily community-involved. Because of the limited pandemic-related data in Saskatchewan, we use a bottom-up approach to highlight the impacts and trends of the pandemic and to compare community experiences against a broader provincial population survey. We have partnered with community-based organizations working in food insecurity, housing precarity and homelessness, mental health, and substance use; a task force formed to harmonize a community response to the pandemic; and public sector unions representing nurses and teachers. Community-based organizations and union leaders joined each project as research partners during the initial project conceptualization and aided in the study design through consultation on the development of the public archive and during the Build Back Better survey development. Community partners have shared their stories for the archive, which aids us in the recruitment of frontline staff and people who access services to participate in oral histories and qualitative interviews. Citizens have shared their stories with us, for instance, about those they lost to COVID. We engage community partners quarterly with formal project updates and further consult as needed as the projects progress. Furthermore, partners will be engaged in community events focused on collaborative analysis and knowledge mobilization.

### Project: Build Back Better

#### Overview

Our health research teams are collecting both quantitative and qualitative data to assess the impacts of the pandemic on 4 interrelated areas: food insecurity, housing precarity and homelessness, mental health, and substance use. Given Saskatchewan’s huge geographical landscape and the importance of rural and northern communities in addition to its 2 major cities, Saskatoon and Regina, and several midsized urban centers, the team also relies on stratified regional analysis to ensure the relevance of the multiple interacting components of this work across the province. The survey also enables an analysis of how access to services has differed for equity-seeking groups across the province. For our study, we define equity-seeking groups as women; Indigenous populations; youth and older adults; 2-spirited, lesbian, gay, bisexual, transgender, and queer/questioning (2SLGBTQ+) persons; immigrants and refugees; those living in remote and rural or underserved areas; and those living with mental health or disabilities. Our mixed methods approach enables the project to connect survey results with qualitative research analyzing the impacts of the pandemic on service provision by affected frontline workers and health, human, and social service organizations.

#### Quantitative Methods

#### Overview

Quantitative data were collected through a population-based cross-sectional web-based survey administered between July and November 2022 (Multimedia Appendix 1). The survey inclusion criteria for the study were the following: (1) a minimum age of 18 years, (2) residing in Saskatchewan, and (3) English language proficiency. We selected the study population for the survey through random probability sampling from the following panels of target populations: the Saskatchewan Community Panel from the Canadian Hub for Applied and Social Research, the Probit Panel from EKOS Research Associates, Inc, and a convenience sample from Voxco’s omnichannel cloud. Quotas were implemented to closely approximate the sample to the Saskatchewan population with respect to gender, age, and geographical location; however, postsurvey weights were also calculated and applied to ensure our study sample aligns with provincial demographics.

The survey data address the following research questions: (1) what is the prevalence of adverse mental health status, substance use, home evictions and housing precarity, and food insecurity in Saskatchewan? How has the prevalence of these challenges changed during the pandemic and compared to that before the pandemic? (2) Has the pandemic differentially affected people in equity-seeking groups (Indigenous groups, visible minority or newcomers, 2SLGBTQ+, people with disabilities, etc) and for those with the greatest social and economic needs (unemployed or underemployed and low-income)?

The web-based survey began with general information about the study, resources for support, and an informed consent form for participation. After providing consent, participants were directed to complete the web-based questionnaire. Participants, on average, took 20 minutes to complete the survey. Upon completion, participants were given the option to enter a draw to win one of fifteen grocery store gift cards valued at CAD $100 (US $73.48) each. The survey included 6 sections: demographics, impacts of the pandemic, food insecurity, housing precarity, mental health, and substance use. Measures included in the survey were taken from or adapted from other validated and reliable instruments to ensure consistency and validity of findings, or they were self-drafted collaboratively by the interdisciplinary team.

#### Demographics

Our survey asked demographic questions to ascertain participants’ age, gender and sexual identity, ethnicity, location, immigrant status, disability, and household composition. We asked respondents about their highest levels of education attained. We also queried whether they are engaged in full-time or part-time employment, unemployed, looking for work or not, stay-at-home parenting, retired, studying, unable to work due to disability, and others. If participants indicated employment, they were asked to specify their area of work for their primary job, which included 22 job areas. We also asked whether participants worked in a frontline position to assess the impacts specific to frontline workers during the pandemic.

#### Impacts of the COVID-19 Pandemic

We measured the impact of the pandemic on participants’ health and well-being (both personal and socioeconomic). First, we assessed the current state of health through self-rated physical and mental health questions, positive COVID-19 tests, the severity and duration of COVID-19 symptoms, and the receipt of COVID-19 vaccines and boosters. Participants’ personal well-being was assessed through self-rated questions on the quality of life and ability to perform day-to-day activities. We also assessed the pandemic’s impact on households’ socioeconomic well-being through 6 indicators drafted by the research team, including changes to individual and household employment, income, household finances, and access to financial or community support. In addition, we measured individual and household coping strategies to mitigate economic challenges through 5 indicators adapted from the Canadian Housing Survey [18].

#### Food Insecurity

We assessed household food insecurity through the Household Food Security Survey Module derived from the Canadian Community Health Survey [19]. Originally developed by the United States Department of Agriculture, the Household Food Security Survey Module is an 18-item standardized and validated module to be used in Canada, consisting of an adult scale (10 items) and a child scale (8 items). These 18 items capture households’ experiences of food insecurity over the 12 months preceding the survey, including the quantity, quality, and affordability of food items. Two other questions were developed to assess the quality of the diet and the accessibility of healthy food during the pandemic.

#### Housing Precarity

Housing precarity was primarily measured by adapting 5 indicators from the Canadian Housing Survey and 8 indicators from the University of California survey of students’ basic needs [18,20]. We asked respondents to select any challenges they experienced managing housing finances (rent and mortgage payments) and whether they were due to the pandemic. The survey also captured respondents’ precarious housing or homelessness experiences by asking about knowing where to sleep, unsafe sleeping arrangements, and shared accommodations such as CouchSurfing or shelter stays. These indicators also included financial challenges to meet housing needs, such as the inability to pay for housing or utilities, taking on debts to pay for housing costs, or cutting back on other expenses to cover housing costs. If an affirmative response was received to any of these questions, a follow-up question was presented to find out if these challenges were partly or entirely due to financial challenges arising from the pandemic. Our survey also included 7 new questions on moving or finding new housing, reasons for moving, including eviction, and if housing challenges arose from the pandemic.

#### Mental Health

We measured participants’ anxiety, depression, stress, and health-seeking behavior for each mental health condition. Anxiety was assessed using the Generalized Anxiety Disorder 7 diagnostic scale, and depression was assessed using the Patient Health Questionnaire 9 scale [21,22]. In addition, we adapted questions from the Mental Health Commission of Canada and the Canadian Centre of Substance Use and Addiction Leger Questionnaire to assess pandemic-driven stressors (18 questions), ability to handle stressful situations (2 questions), and overall mental health (1 question) [21]. Finally, drawing on questions from the Mental Health Commission of Canada, the Canadian Centre of Substance Use and Addiction Leger Questionnaire, and the Mental Health Research Canada’s Mental Health and COVID Tracker poll, we assessed participants’ health-seeking behavior through their access to and attitudes about opting for treatments or supports for mental health concerns [21,22]. Questions included whether participants sought treatment from a trained professional, the ease (or difficulty) of accessing these services and reasons for not accessing them, mental health diagnoses from a medical or psychological professional, and the time of receiving a diagnosis. We also included 2 self-drafted questions about participants’ feelings about needing help with mental health and for what purpose they sought help or treatment.

#### Substance Use

We measured participants’ substance use in 5 categories: alcohol, tobacco or nicotine, cannabis, prescription medication (opioids, sedatives [benzodiazepines and non-benzodiazepines] and barbiturates, stimulants, and cannabis), and other drug or substance use; self-rated impacts of the pandemic on use of each substance; and substance use concerns, treatments, and supports. We assessed participants’ use of each substance using indicators either taken or modified from the Mental Health Commission of Canada and the Canadian Centre on Substance Use and Addiction Leger Questionnaire [21], the Canadian Alcohol and Drugs Survey [23], and the Canadian Nicotine and Tobacco Survey [24] or were self-drafted. We asked about frequency, quantity, and preferences or modes of use for each substance category, where applicable, and these indicators were primarily taken or adapted from the above instruments. We measured the self-rated impacts of the pandemic on substance use through self-drafted questions. We modified an indicator from the Mental Health Commission of Canada and the Canadian Centre on Substance Use and Addiction Leger Questionnaire to measure self-rated concern of use for each substance. If participants indicated some level of concern for one or more of the five substance categories, we asked about seeking treatment or support services from a trained professional, the ease (or difficulty) of accessing these services, reasons for not accessing services, substance use disorder diagnoses from a medical or psychological professional, and when a diagnosis was received.

Following the closing of the survey, we checked and cleaned the data for outliers to identify if it was skewed in a certain direction. We checked variables with a significant proportion of missing values to see if there was any systematic pattern in the missing data. We conducted a multiple imputation analysis and imputed data for the following variables: location of the residence (5.4%), sex (1.6%), age (3.2%), ethnicity (2.4%), education (3.0%), employment status (1.8%), income (7.1%), and housing insecurity (13.7%).

The final data set, deidentified and cleaned, was shared with the core research team members. Preliminary analyses have been conducted to identify the impact of the pandemic on the overall study population. We presented the findings as frequencies and percentages for categorical variables with associated confidence intervals and means and standard deviations for continuous variables. In addition, we use graphs and maps to present our findings to community partners for ease of understanding key results. Next, we will perform multivariable linear and logistic regression analyses to investigate the risk factors that have intensified households’ experiences of food insecurity, housing precarity and homelessness, mental health, and substance use.

#### Qualitative Methods

The surveys help to provide important quantitative indicators of a broader range of impacts of the pandemic on other public health services, but we also recognize the need to connect the numbers with the stories of how people have experienced this historical moment. We are collecting qualitative data to answer the following research questions: (1) what role have frontline human services agencies played in helping people deal with the challenges of the pandemic? What barriers have hindered access to services? What makes services effective? (2) What do people who access services and service providers believe the priorities for policy and practice implementation should be in the 4 interrelated study areas?

We are collecting data through open-ended interviews with two categories of respondents: (1) service providers from partner agencies (Multimedia Appendix 2) and (2) people who access services within partner agencies (Multimedia Appendix 3). For service providers, we will interview 5 service providers working in agencies in the 4 focus areas (n=20). For people who access services, we will interview 8-10 people per focus area (n=24-32). Interviews last between 30 and 60 minutes, and we provide an honorarium of CAD $25 (US $18.37) to people who access services at participating agencies to compensate for their time and contribution to the project. Service providers will not receive an honorarium as their interviews will be related to their employment at partner agencies, and thus they will be compensated for their time through their workplaces. After the initial interviews, we will consult partner organizations to expand participant recruitment in Regina, midsized urban centers, and rural regions in the province.

Interviews take place in locations that are best suited to individual participants in one of 3 options: in-person at partner or host organizations from which they were recruited; remotely over Zoom, where participants decide where is most advantageous; or remotely over Zoom at partner or host organizations from which they were recruited. Not all people who access services have access to technology for virtual interviews, and some are not comfortable doing interviews in person; thus, we work with host organizations to facilitate an ideal setting for each participant. To further ensure the safety of participants, interviewers completed Tri-Council Policy Statement: Ethical Conduct for Research Involving Humans certification and will complete trauma-informed care training prior to recruiting people who access services.

The analysis of qualitative interview data will begin with the first interview and continue throughout the data collection period. Both inductive and deductive methods of data coding will be used. Reflection on the quantitative data and engagement with the community partners will help us identify the initial coding framework. We will use thematic analysis to identify initial codes in the first rounds of data analysis across the 4 focus areas and begin conceptualizing broad themes [25]. Subsequent iterative rounds of analysis will sharpen our coding into more specific themes. We will also be analyzing for saturation and adapting and updating codes to ensure extracted data reflect participants’ contributions as interviews progress. Moreover, we will consult community partners throughout the process. For the data retrieved from people who access services, we will extract common themes across the 4 focus areas as well as stratified thematic analysis for themes specific to equity-seeking groups and other characteristics as the research team identifies emerging themes. For service providers, we will extract themes across the 4 focus areas as well as a broader thematic analysis illuminating common experiences faced by those working in human services during the pandemic.

We use both quantitative and qualitative data to gain a broad understanding of the impact of the COVID-19 pandemic on people’s lives and well-being in Saskatchewan. The quantitative data help us identify specific groups of people and areas of concern that are generalizable to the province and comparable with national data sources, and the qualitative data helps to provide a more granular level of detail about impacts on individuals. The qualitative approach allows us to emphasize the intersectionality of the pandemic and other public health needs that are not necessarily readily parsed into categories when it comes to how they were experienced in real time. By allowing participants to describe their encounters in their own words, we seek to enrich our understanding of qualities that include elements of pacing or timing of decisions, to better understand how providers and clients made decisions that prioritized health and economic needs in real time, and how they reflect on the impact of the pandemic and government policies on meeting their basic needs. In combining quantitative and qualitative approaches, we enhance our understanding of the wider impacts of the pandemic, generating a wide range of evidence that is crucial for policymaking in postpandemic recovery.

### Project: Saskatchewan’s COVID-19 Public Archive

Historians on the team are collecting and analyzing a large archive of academic, oral history, and social and digital media sources to develop a unique digital community archive. The project began as a response to the concern that COVID-19 risked being another forgotten pandemic because of the ephemeral nature of web-based primary sources. We have expanded the project to incorporate new digital methods to increase the capacity to collect digital materials and lay the foundation for writing a social history of the COVID-19 pandemic in Saskatchewan [26]. We began in March 2020 by setting up an Archive-It repository to automatically record a web archive of government, organizations, and media reports related to the COVID-19 pandemic in Saskatchewan. We took inspiration from other jurisdictions engaged in COVID-19 archiving and drew from examples of archives established to capture “disaster responses” that include public memorialization to remember events like Hurricane Katrina in the United States and the 9-11 attacks [27]. Remembering—including analyzing and learning from—COVID-19 requires new theoretical and methodological approaches that capture and preserve the experience for future generations.

We began collecting oral histories in 2020, interviewing local business owners, reporters, and a prominent retired doctor. The new funding and formal partnerships with community organizations allowed us to expand the interviews in the summer of 2022 as we collected perspectives from vaccine scientists, intensive care unit doctors and nurses, frontline workers, employees at a safe consumption site and food banks, justice-involved individuals, and other marginalized racial and ethnic groups of residents. Interviews are conducted using an open-ended interview guide. We plan to expand the scope of these interviews, both by supporting the qualitative research interviews led by the Build Back Better team with at-risk populations and by continuing to seek out oral history interviews with as wide a range of people as possible with unique experiences during the pandemic.

Exploring the Archive-It collection in partnership with the Archives Unleashed project [28], we quickly recognized that we had captured the important institutional experiences via their websites in the province but missed a lot of the social media discussion. We are working to archive large sections of Twitter and Facebook to preserve this public debate. Twitter’s application programming interface provides free access to academics, allowing us to record millions of tweets related to the pandemic starting in 2020. We collected every post made by the premier of Saskatchewan during the pandemic, along with all the replies to his tweets, the conversations using the #COVID19SK hashtag, and conversations involving numerous other COVID-19 influencers in the province. Facebook does not provide an application programming interface and actively blocks web crawling software. As a result, it requires manual scrolling of content on the platform. We, therefore, restricted our archiving efforts to focus on the public Facebook page and public posts from a small number of influential accounts commenting on the public health measures. One of these accounts has since been removed from the platform, reminding us again of the ephemeral nature of this material.

Finally, while the Archive-It collection holds thousands of web pages from news outlets, they are not cataloged and are difficult to navigate. To create a more user-friendly repository, we are using citation database software Zotero (Corporation for Digital Scholarship, George Mason University) to catalog stories on the pandemic in Saskatchewan. The collection includes 3200 stories and hundreds of documents generated by community-based organizations responding to community needs during the pandemic. Research assistants work on assigned topics, including homelessness, substance use, nursing, or the start of the COVID-19 pandemic in Saskatchewan. Our research assistants clean the metadata and add keyword tags, including the people mentioned in the news stories, the places discussed, and the main topics and periods of the pandemic, such as “Fourth Wave.” The tag categories are carefully managed and edited to avoid using synonyms or the proliferation of tags to the point they become useless. The Zotero database, which includes the HTML code from the original stories, provides another means to preserve web-based content and creates opportunities for text-mining approaches that will help us navigate the much larger Archive-It collection.

### Ethics Approval

This study is approved by the Research Ethics Board at the University of Saskatchewan (Beh-1945) and is being conducted in accordance with the guidelines and stipulations of the Tri-Council Policy Statement: Ethical Conduct for Research Involving Humans [29]. Changes to the protocol will be submitted for approval through amendment requests to the Research Ethics Board. Participation in either project is completely voluntary.

## Results

The “Build Back Better” and “Saskatchewan’s COVID-19 Public Archive” projects received federal funding in March and April 2022, respectively. Survey data were collected between July and November 2022 and are being analyzed with results expected to be published in the fall of 2023. Qualitative interview data were collected between March and June 2023, and 19 interviews have been completed. Qualitative data are being analyzed, with results expected to be published in winter 2024. Initial oral histories and archival data collection began in June 2022 and will conclude in the summer of 2023. Thirty oral histories have been completed. The archive will be continuously updated. On the 3-year anniversary of the first COVID-19 case in the province, we released a new feature on the website: a digital memorial to the lives lost to COVID-19 called “Remember Lives Not Numbers.” After reviewing publicly accessible obituaries and media stories about COVID-19 deaths in the province, we created a web-based memorial featuring over 100 entries that honor the lives lost in the province due to COVID-19. This digital memorial includes a public portal, which allows for continued submissions to the site to help expand the reach of this memorial to the hundreds of cases that were not covered in the news media or explicitly mentioned in published obituaries.

## Discussion

The global COVID-19 pandemic has been a significant crisis that catalyzed both immediate and long-term changes, prompting collective responses across and within nations, the effects of which are still unfolding (in May 2023). The Remember Rebuild Saskatchewan project has a wide impetus to illuminate what we have learned—both what went wrong and what worked well in our collective responses—and to preserve this legacy for future health crises and events. Our innovative and collaborative program of research harnesses community-engaged research and showcases the strengths of different health, social sciences, and humanities disciplines, and we will produce relevant and accessible quantitative and qualitative findings.

Within “Build Back Better,” we expect findings from our general population survey to illuminate disparities in outcomes in the 4 focus areas (food insecurity, housing precarity, mental health, and substance use) in Saskatchewan, where groups such as youth, newcomers, Indigenous peoples, 2SLGBTQ+ individuals, people with low income, or people living with disabilities experience higher rates of negative outcomes. In addition, we expect to elucidate the dual experiences of outcomes during the COVID-19 pandemic—namely, poor mental health and problematic substance use, housing precarity, and food insecurity—which are often experienced together, adding complexity and vulnerability to people’s lives.

Our survey design limits our understanding of the depth and intensity of the disruption and negative effects caused by the pandemic, though it captures well the prevalence, scope, and factors associated with these negative effects. We expect our qualitative interview analysis to reveal the depth and intensity of the disruption and negative effects. First, service-providing organizations in Saskatchewan were greatly impacted by the pandemic through modified service provision in accordance with public health guidelines and increased service demand and usage in each of the 4 focus areas. Additionally, we expect interview analysis to reveal that people accessing services in Saskatchewan faced increased barriers to accessing services, such as less access due to shutdowns and public health restrictions, which exacerbated negative health outcomes across the 4 focus areas.

Our COVID-19 public archive is a legacy product and will host the findings from all aspects of our research and outreach, as well as maintain a voluntary submission feature. The Remember Rebuild Saskatchewan findings exist in a freely accessible web-based repository, available on the project website [30]. Creating a public archive enables us to recognize the diversity of the pandemic experience and provides the foundation for future research on the changes we need to implement to build resilience as we prepare for a future with more public health crises. Findings will be available in various formats, such as academic knowledge translation outputs from Build Back Better, the oral his or her or their-stories of various Saskatchewan residents, social and digital news media stories, and audiovisual and creative contributions such as artwork or music. The archive, inclusive of the different components of each project, will be promoted widely to the public at a community event through collaboration with the Saskatoon Public Library in early 2024 and interviews with digital and social media journalists. These events help raise awareness of the projects in addition to facilitating contributions to the archive as the collection continues. We have prepared media releases and policy briefs to further engage the public, media, and policy makers.

We will compile project findings into various academic outputs, including journal articles, research posters, technical reports, conference presentations, and infographics, which will also be stored in the archive. We will additionally host academic contributions from other pandemic-related projects from the Remember Rebuild Saskatchewan team and other research contributions on the province’s COVID-19 experience beyond those from the team. In this way, the archive will also act as a research hub for the pandemic in Saskatchewan, offering a repository for data and evidence documenting the multifaceted impacts of the pandemic in Saskatchewan.

## Appendix

Multimedia Appendix 1 Build Back Better Questionnaire.

Multimedia Appendix 2 Build Back Better--Service provider interview guide.

Multimedia Appendix 3 Build Back Better--People accessing services interview guide.

Multimedia Appendix 4 Peer review report (CIHR).

## Abbreviations

2SLGBTQ+: 2-spirited, lesbian, gay, bisexual, transgender, and queer/questioning

## Data Availability

Data and stories collected in this work are archived for preservation and freely accessible on the Remember Rebuild Saskatchewan website [30].

## References

[1] (2023). COVID-19 epidemiology update: summary. Government of Canada.

[2] (2021). Economic impacts of COVID-19 in the provinces and territories. Statistics Canada.

[3] Djuric M (2022). ‘It’s time’: Moe says COVID-19 restrictions on freedoms will soon end in Saskatchewan. Toronto Star.

[4] Shield D (2021). Sask. Epidemiologist concerned about potential easing of COVID-19 restrictions. CBC News.

[5] Charlton J (2022). Sask. COVID-19 dashboard goes dark in transition to 'living with COVID'. CTV News.

[6] (2022). Our impact. Regina Food Bank.

[7] (2021). Saskatoon Food Bank and Learning Centre (SFBLC). 2022 Annual Report: City of Bridges. issuu.

[8] Annual report SFBLC 2021. Saskatoon Food Bank and Learning Centre.

[9] Latimer K (2022). Housing advocates concerned by high number of homeless people in Saskatoon. CBC News.

[10] (2022). Point in time count 2022. Saskatoon Housing Initiatives Partnership (SHIP).

[11] Docherty A 2021 Regina homelessness count. Flow Community Projects.

[12] Tarasuk V, Li T, Fafard St-Germain AA (2022). Household food insecurity in Canada, 2021. Proof.

[13] Muhajarine N, Adeyinka DA, Pisolkar V, Ahmed MS, Kallio N, Coomaran V, McIntosh T, Novik N, Jeffery B (2022). Equity analysis of repeated cross-sectional survey data on mental health outcomes in Saskatchewan, Canada during COVID-19 pandemic. Int J Environ Res Public Health.

[14] (2021). Taking the pulse of COVID-19 in Saskatchewan—in 6 charts. Saskatchewan Population Health and Evaluation Research Unit (SPHERU).

[15] (2022). Are the kids alright? Special issue: taking the pulse of COVID-19 in Saskatchewan—in 7 charts. Saskatchewan Population Health and Evaluation Research Unit (SPHERU).

[16] (2022). Taking the pulse of COVID-19 in Saskatchewan—in 13 charts. Saskatchewan Population Health and Evaluation Research Unit (SPHERU).

[17] (2023). Confirmed and suspected drug toxicity deaths (2016 - most recent). Saskatchewan.

[18] (2022). Canadian housing survey. Statistics Canada.

[19] (2023). Canadian community health survey - annual component (CCHS). Statistics Canada.

[20] Institutional research and academic planning. University of California.

[21] (2021). Mental health and substance use during COVID-19. Leger.

[22] National poll on Canadian mental health: 14 studies in an ongoing series. Mental Health Research Canada (MHRC).

[23] (2021). Canadian alcohol and drugs survey (CADS). Statistics Canada.

[24] (2022). Canadian tobacco and nicotine survey (CTNS). Statistics Canada.

[25] Braun V, Clarke V (2006). Using thematic analysis in psychology. Qual Res Psychol.

[26] Jones EW, Sweeney S, Milligan I, Bak G, McCutcheon JA (2021). Remembering is a form of honouring: preserving the COVID-19 archival record. FACETS.

[27] Cohen D, Rosenzweig R (2006). Digital History: A Guide to Gathering, Preserving, and Presenting the Past on the Web.

[28] (2020). COVID19 Saskatchewan. Archive-It.

[29] (2020). Tri-council policy statement: ethical conduct for research involving humans - TCPS-2 (2018). Government of Canada. Panel on Research Ethics.

[30] (2023). Remember Rebuild Saskatchewan. Remember Rebuild SK.

