# Supplementary material for: Capturing and Documenting the Wider Health Impacts of the COVID-19 Pandemic Through the Remember Rebuild Saskatchewan Initiative: Protocol for a Mixed Methods Interdisciplinary Project
Source: JMIR Res Protoc. 2023 Jun 6;12:e46643. doi: 10.2196/46643 (PMC10282902; doi:10.2196/46643)
Supplement: Multimedia Appendix 1 [file resprot_v12i1e46643_app1.pdf]

Muhajarine N, Dixon J, Dyck E, Clifford J, Chassé P, Gupta SD, Christopherson-Cote C, Remember Rebuild Saskatchewan Team. Capturing and Documenting the Wider Health Impacts of the COVID-19 Pandemic Through the Remember Rebuild SaskatchewanInitiative: Protocol for a Mixed Methods Interdisciplinary Project JMIR Res Protoc 2023;12:e46643. URL: https://www.researchprotocols.org/2023/1/e46643/
doi: 10.2196/46643.

Do you live in Saskatchewan?

- ○ Yes
- ○ No

SCREEN2

Please have one adult (aged 18 or older) in your household complete this survey. If there are two or more adults in your household, choose the adult who is most often responsible for managing the grocery shopping or household finances. If two or more persons share these responsibilities, either may respond to the survey.

DESCRIPTION

UNIVERSITY OF SASKATCHEWAN

University of Regina

Participant Consent Form

You are invited to participate in a research study entitled: Build Back Better: COVID-19 in Saskatchewan

Researchers:
We are a team from the Universities of Saskatchewan and Regina and community-based organizations, led by Principal Investigators:

Dr. Nazeem Muhajarine,
Professor, Department of Community Health and Epidemiology
College of Medicine, University of Saskatchewan
Director, Saskatchewan Population Health and Evaluation Research Unit (SPHERU)
t: 306-966-7940 | e: nazeem.muhajarine@usask.ca

Dr. Erika Dyck, Department of History

Professor, Canada Research Chair in the History of Medicine

College of Arts & Science, University of Saskatchewan

t: 306-966-5049 | e: erika.dyck@usask.ca

To learn more about the rest of the research team, click here.

Background:
You are invited to participate in Build Back Better: COVID-19 in Saskatchewan, a study to help us understand how the COVID-19 pandemic has affected the lives of people in Saskatchewan in four areas: food security, housing, mental health, and substance use. Whatever the pandemic has been like for you—good or bad, a big impact or not much change—we want to know about it.

Our team is made up of researchers, health providers, and community leaders committed to understanding how the pandemic was experienced across communities. Gathered feedback will inform policy decisions that helps everyone get the care that they need in Saskatchewan.

Please have one adult in your household complete this survey. If there are two or more adults in your household, choose the adult who is most often responsible for managing the grocery shopping or household finances. If two or more persons share these responsibilities, either may respond to the survey.

Confidentiality and potential risks:

Participation in this research is voluntary, and you can stop the survey at any time. We encourage you to complete the entire survey, but you may skip any question by hitting the "next" button to advance to the next page of the survey. You may withdraw from the survey by closing your internet browser.

Once you have finished the survey and clicked on "submit," you will no longer able to withdraw, **because your responses are anonymous, and we won't know which answers are yours.**

You cannot be personally identified based on the data we collect. The survey is hosted by Voxco, a Canadian-owned and managed company whose data are securely stored in Canada (see Voxco's privacy policy here).

Taking part in this study is unlikely to cause any harm to you but if you need support: If you have a medical or mental health emergency, call 911 or go to the nearest open clinic or emergency room. If you are concerned that you and/or your child are a danger to yourself and/or themselves or to others, call 911 or go to the nearest open clinic or emergency room.
connect with a health and/or mental health care provider (e.g., doctor, nurse practitioner, counsellor, social worker, psychiatrist, etc.).
call 8-1-1 for confidential, free, 24-hour health and mental health and substance use advice, education, and support telephone line available to the people of Saskatchewan, staffed by experienced and specially trained Registered Nurses, Registered Psychiatric Nurses, and Registered Social Workers.
use **resources** available to you online or by phone: https://rememberrebuildsk.ca/resources
If you have a medical or mental health emergency, call 911 or go to the nearest open clinic or emergency room. If you are concerned that you and/or your child are a danger to yourself and/or themselves or to others, call 911 or go to the nearest open clinic or emergency room.

This study is part of a larger research project—Remember and rebuild: health equity lessons from Saskatchewan's COVID-19 experience (Beh-1945)—and has been reviewed and approved on ethical grounds by the University of Saskatchewan Research Ethics Board. Any questions regarding your rights as a participant may be addressed to that committee through the Research Ethics Office: ethics.office@usask.ca (306) 966-2975. Out of town participants may call toll free (888) 966-2975.

This study is funded by the Canadian Institutes for Health Research (CIHR) and the Social Sciences and Humanities Research Council (SSHRC), For more information on the study itself please email remember.rebuildsk@usask.ca.

By clicking on "next" and answering the questions that follow, you are saying that you freely agree to take part in the study and that you understand what you have just read about how the study works.

INTRO

We will begin the survey by asking some general questions about how the pandemic may have affected you. Please note that when we use the word “COVID” we mean being sick with or testing positive for COVID-19 (coronavirus). When we use the word “pandemic” we mean all the changes related to COVID since March 2020, like having to wear a mask, physical distancing, and not being able to do certain activities.

We are interested in hearing about your experiences. There are no right or wrong answers and your responses will never be linked back to your personally.

IC2

In general, how is your physical health now?

- ○ Excellent
- ○ Good
- ○ Average
- ○ Poor
- ○ Very Poor

IC1

Thinking about yourself now, as compared to before the pandemic began (March 2020), how would you describe any change you've experienced in the following:

	Much worse now	Somewhat worse now	About the same	Somewhat better now	Much better now
Your life overall	○	○	○	○	○
Your physical health	○	○	○	○	○
Your ability to handle unexpected and difficult situations	○	○	○	○	○
Your ability to handle day-to-day demands	○	○	○	○	○

[illegible] in your life					

IC6

Have you ever tested positive on a PCR test or a Rapid Antigen Test?

- ○ No
- ○ Yes, once
- ○ Yes, more than once

IC7

Have you ever had symptoms you were pretty sure meant you had COVID even though you didn’t test positive?

- ○ No
- ○ Yes

IC8

How sick were you?

- ○ No symptoms
- ○ Mild to moderate symptoms but not hospitalized
- ○ Hospitalized

IC9

If you had COVID <u>more than one month ago</u>, are you still experiencing symptoms you believe may be related to COVID, such as tiredness, shortness of breath, difficulty concentrating or remembering, cough, fast or pounding heartbeat?

- ○ No
- ○ Yes
- ○ Maybe
- ○ Had COVID less than one month ago

IC10

Have you been vaccinated against COVID-19?

- ○ No
- ○ Yes, received one dose of a vaccine
- ○ Yes, received two doses of a vaccine
- ○ Yes, received two doses and 1 booster dose as well
- ○ Yes, received two doses and 2 booster doses as well

IC11

How likely are you to get a COVID-19 vaccine sometime in the future?

- ○ Very likely
- ○ Somewhat likely
- ○ Somewhat unlikely
- ○ Very unlikely

IC12

How likely are you to get another dose of a COVID-19 vaccine sometime in the future?

- ○ Very likely
- ○ Somewhat likely
- ○ Somewhat unlikely
- ○ Very unlikely

IC13

What do you think of the provincial government's handling of the pandemic overall?

- ○ Excellent
- ○ Good
- ○ Average
- ○ Poor
- ○ Very poor

IC14

In its efforts to control the pandemic, do you think the provincial government...

- ○ Did too much?
- ○ Did too little?

INTRO_FINANCE

Next are some questions about your household and how the pandemic may have affected your household's finances. By <u>household</u> we mean you and whoever else usually lives with you.

If you and other people you live with DO NOT share income and expenses (for example, you pay your own share of rent and buy your own food), please answer the questions about household finances based on your own situation.

FS1A

How many people are currently living in your household, including yourself?

[ ]

- ○ N/A
- ○ Refusal

○ Do not know

FS1B

Of these people, how many are under the age of 18?

Please put 0 if there are no children in the household.

○ N/A
○ Refusal
○ Do not know

FS1C

Did the number of people living your household change since March 2020?

○ Yes, the number of people increased
○ Yes, the number of people decreased
○ No, the number of people stayed the same
○ Other

IC15

Which of the following best describes your current employment status?

- ○ Employed full-time
- ○ Employed part-time
- ○ Unemployed – looking for work
- ○ Unemployed- not looking for work
- ○ Staying home raising children full-time
- ○ Retired
- ○ Student (full/part time)
- ○ Unable to work due to disability
- ○ Other (please specify) ______

IC17

In which of these areas do you work?

If you have more than one job, please choose the one that you do most often.

- ○ Accommodation, hospitality, and food service
- ○ Activities of households as employers
- ○ Administrative and support service
- ○ Agricultural, forest, and fisheries
- ○ Arts, entertainment, and recreation
- ○ Construction
- ○ Education
- ○ Energy
- ○ Financial and insurance
- ○ Human health and social work
- ○ Information and communication
- ○ International organizations, diplomacy, associations
- ○ Manufacturing
- ○ Mines and quarries
- ○ Other service activities
- ○ Professional, scientific, and technical
- ○ Public administration and defence, compulsory social security
- ○ Real estate
- ○ Transport and storage
- ○ Water supply, sanitation, waste management, and remediation
- ○ Wholesale and retail trade

○ Other (please specify) ______

IC18

<u>At any time during the pandemic</u>, were you a frontline worker?

By this we mean that you were often in contact with other people (more than just a few co-workers), for example, nurse or other health care worker, teacher/teacher assistant, salesperson, construction worker, or restaurant worker.

○ Yes

○ No

IC19

Are you working at the same job now?

○ Yes

○ No, but still a frontline worker

○ No, and not a frontline worker now

IC20

Has your employment or income situation changed as a result of the pandemic at any time since March 2020? Please check all that apply.

- ☐ My job situation has not changed
- ☐ I am now working from home full-time
- ☐ I am now splitting my time between going in to work and working from home
- ☐ I was working from home and am now working at my workplace
- ☐ I was laid off
- ☐ My hours were reduced
- ☐ My hours were increased
- ☐ My rate of pay decreased
- ☐ My rate of pay increased
- ☐ I was never laid off or terminated but I have started a new job
- ☐ I quit my job due to COVID-19 concerns
- ☐ Other (please specify) ______

IC21

Did your household finances change during the pandemic?

- ○ Got a lot worse
- ○ Got a little worse
- ○ No change
- ○ Got a little better
- ○ Got a lot better

IC22

Is this because of the difficulties arising from the pandemic?

- ○ Yes
- ○ Partly
- ○ No

IC23

Did you have to use some of your savings to cope with the impacts of the pandemic?

- ○ Yes
- ○ No
- ○ I do not have savings

IC24

Have you or any member of your household received any financial assistance from the government to cope with the difficulties arising from the pandemic?

If you don’t know about other household members, just answer for yourself.

- ○ Yes
- ○ No

IC25

Which of the following types of financial assistance did you receive? Check all that apply.

- ☐ Canada Emergency Response (CERB)
- ☐ Canadian Emergency Student Benefit (CESB)
- ☐ Employment Insurance (EI)
- ☐ Saskatchewan Income Supplement (SIS)
- ☐ Saskatchewan Assured Income for Disability (SAID)
- ☐ Saskatchewan Assistance Program (SAP)
- ☐ First Nations band or other Indigenous government financial support (including the Indigenous Community Support Fund)
- ☐ Employer financial support
- ☐ Other (please specify) ______

IC26

Have you or any member of your household received any practical help (such as grocery gift cards, meals from a soup kitchen or food hampers) from a non-governmental organization, charity, food bank, or religious organization <u>since the pandemic started (March 2020)?</u>

- ○ Yes
- ○ No

IC27

Was it due to need arising from the pandemic?

- ○ Yes
- ○ Partly

○ No

IC28

<u>In the past 12 months</u>, did any member of your household ever do any of the following because they were short of money due to the pandemic?

	No	Yes, sometimes	Yes, often
Ask for financial help from friends or relatives for day-to-day expenses	○	○	○
Take on debt or pawn something to help with day-to-day expenses	○	○	○
Sell belongings to help with day-to-day expenses	○	○	○

IC29

<u>In the past 12 months</u>, how difficult or easy was it for your household to find enough money for necessary expenses like food, housing, transportation, and medicine?

○ Very difficult

○ Difficult

○ Neither difficult nor easy

○ Easy

○ Very easy

IC30

Is this because of the difficulties arising from the pandemic?

○ Yes

○ Partly

○ No

IC31

Thinking about the next six months, how concerned are you about your household's ability to pay for everything you need?

○ Very concerned

○ Somewhat concerned

○ Slightly concerned

○ Not at all concerned

IC32

What is your best estimate of the total yearly income received by all household members, from all sources, before taxes and deductions?

(Income can come from work, government aid, pensions or investments. Examples include Employment Insurance, Social Assistance, Child Tax Benefit, maternity/paternity leave, and other income such as child support, alimony and rental income. If you do not share income and expenses with other people you live with, please report your own income.)

○ Less than $20,000

○ $20,000-$39,999

○ $40,000-$69,999

○ $70,000 or more

○ Don't know

INTRO_FOOD

We know that one of the areas of life the pandemic affected for many people is the way they eat. We would like to know more about how your eating, cooking and your ability to get food may have been affected.

Please be as honest as possible and know that your answers will never be linked back to your personally.

FS1

At any time during the pandemic, did any of these apply to you? Please check all that apply.

- ☐ Ate less fruits and vegetables
- ☐ Ate more fruits and vegetables
- ☐ Ate more salty or sugary snack foods or candy
- ☐ Ate less or skipped meals
- ☐ Cooked more
- ☐ Got takeout food from restaurants more often
- ☐ It was harder than usual to get culturally appropriate or traditional food
- ☐ Food I preferred to eat was less available in my community
- ☐ I did not have transportation to get the food I wanted
- ☐ I grew my own food/gardened (e.g. backyard, community, or container gardening)
- ☐ Other (please specify) ________

FS2

Overall, how much was your household's ability to get enough healthy food to eat affected by the pandemic?

- ○ A lot
- ○ A little
- ○ Not at all

FSC

This section is about the food situation for your household <u>in the past 12 months</u>. Please tell us how often each of these things has been true for you.

	Never true	Sometimes true	Often true
You worried that food would run out before you got money to buy more.	○	○	○
The food that you bought just didn't last, and there wasn't any money to get more.	○	○	○
You couldn't afford to eat balanced meals.	○	○	○
You relied on only a few kinds of low-cost food to feed the child of your household/family because you were running out of money to buy food.	○	○	○
You couldn't feed the child a balanced meal, because you couldn't afford it.	○	○	○

FSC_035

<u>In the past 12 months</u>, has your child not eaten enough because you or other adult members just couldn't afford enough food?

○ Never true

○ Sometimes true

○ Often true

FSC2

The following few questions are about the food situation <u>in the past 12 months</u> for you or any other adults in your household.

	No	Yes
Did you or other adult household members ever cut the size of your meals or skip meals because there wasn’t enough money for food?	○	○
Did you personally ever eat less than you felt you should because there wasn’t enough money to buy food?	○	○
Were you personally ever hungry but didn't eat because you couldn't afford enough food?	○	○
Did you personally ever lose weight because you didn't have enough money for food?	○	○

FSC_045

<u>In the past 12 months</u>, how often did you or other adult household members cut the size of your meals or skip meals because there wasn’t enough money for food?

- ○ Almost every month
- ○ Some months but not every month
- ○ Only 1 or 2 months

FSC_065

<u>In the past 12 months</u>, did you or any other adult member of the household ever not eat for a whole day because there wasn't enough money for food?

- ○ Yes
- ○ No

FSC_070

How often did this happen?

- ○ Almost every month
- ○ Some months but not every month
- ○ Only 1 or 2 months

FSC2_075

Now, a few questions on the food experiences for children in your household. The following questions are about the food situation in the past 12 months.

	No	Yes
Did you or any other adult members of your household ever cut the size of a child’s meals because there wasn't enough money for food?	○	○
Did any child in your household ever skip meals because there wasn't enough money for food?	○	○
Was any child ever hungry but you just couldn't afford more food?	○	○
Did a child in your household not ever eat for a whole day because there wasn't enough money for food?	○	○

FSC_085

How often did any child in your household skip meals because there wasn't enough money for food?

- ○ Almost every month
- ○ Some months but not every month
- ○ Only 1 or 2 months

INTRO_HOUSING

Now, we would like to ask about your housing situation.

HS1

In the past 12 months, did you move to a different home?

- ○ Yes
- ○ No

HS2

Was this because of difficulties arising from the pandemic?

- ○ Yes
- ○ No

HS3

How many times have you moved in the past 12 months?

- ○ N/A
- ○ Refusal
- ○ Do not know

HS4

How many rooms are in your current home? (Rooms means bedrooms, living rooms and dining rooms).

- N/A
- Refusal
- Do not know

HS5

How many bedrooms are there?

- N/A
- Refusal
- Do not know

HS6

Is your current home owned by a member of your household?

- Yes
- No

HS9

In the past 12 months, has your household ever skipped or delayed a mortgage or rent payment?

- Yes
- No

HS10

Was it due to financial difficulties arising from the pandemic?

- ○ Yes
- ○ No

HS11

Did your skipping or delaying a mortgage or rental payment in the past 12 months result in any of the following situations?

- ○ Selling your home
- ○ Foreclosure or Power of Sale
- ○ Forced/had to move
- ○ Other (please specify) ______
- ○ None of the above

HS11A

Which of the following best describes how you were forced to move?

- ○ Formal (landlord got an eviction order)
- ○ Informal (You had to leave due to actions by landlords even without an eviction order)

HS11B

What was the reason you were forced to move?

- ☐ Increase in rent
- ☐ Threat of taking formal procedure by landlords
- ☐ Unable to pay rent or utilities
- ☐ Other (please specify)

HS12

What proportion of total household income is usually spent on total housing costs (rent/mortgage payment including utilities and insurance)?

If you do NOT share your income and expenses with the other people you live with, please tell us how much of YOUR income is spent on YOUR share of housing costs.

- ○ Less than 30%
- ○ 30 – 50%
- ○ More than 50%
- ○ I don’t know

HS_

Now, we will ask a few more questions about housing in the past 12 months.

	No	Yes
Did you ever not know where you were going to sleep at night, even for one night?	○	○
Was there a time when you had to sleep somewhere unsafe?	○	○
Was there a time when you had to sleep outside, stay with friends or relatives (couch surf) (or stay in a shelter?	○	○
Did you have to move in with others due to financial problems?	○	○
Were you ever unable to pay the full amount of utilities such as gas, internet etc.?	○	○
Did you have to sublease your place because your income was not enough to cover your costs?	○	○
Did you take on credit card debt to pay for housing?	○	○
Did you have financial help from friends or relatives to pay for housing?	○	○
Did you have to cut back on other expenses (such as food/medication/dental care) in order to cover housing costs?	○	○

HS_I

You indicated you experienced these issues in the past 12 months. Was it due to financial difficulties arising from the pandemic?

	No	Partly	Yes
Not knowing where you were going to sleep at night, even for one night	○	○	○
You had to sleep somewhere unsafe	○	○	○
You had to sleep outside, stay with friends or relatives (couch surf) (or stay in a shelter	○	○	○
You had to move in with others due to financial problems	○	○	○
You were unable to pay the full amount of utilities such as gas, internet etc.	○	○	○
You had to sublease your place because your income was not enough to cover your costs	○	○	○
You took on credit card debt to pay for housing	○	○	○
You had financial help from friends or relatives to pay for housing	○	○	○
You had to cut back on other expenses (such as food/medication/dental care) in order to cover housing costs	○	○	○

INTRO_MH

We are interested in finding out how the pandemic may have affected your mental health.

By "Mental health" we mean:
***Your feelings--how often you feel sad, scared or worried and whether you get upset or mad easily,**
***How well you cope with problems, and**
***How well you get along with other people.**

MH1

In general, how is your overall mental health now?

- ○ Very good
- ○ Good
- ○ Average
- ○ Poor
- ○ Very poor

MH2

How would you say your mental health is now, <u>compared to before the pandemic began (March 2020)</u>?

- ○ Much better now
- ○ Somewhat better now
- ○ About the same
- ○ Somewhat worse now
- ○ Much worse now

MH3

Overall, how do you think the pandemic affected your mental health?

- ○ Made it much better
- ○ Made it somewhat better
- ○ No effect
- ○ Made it somewhat worse
- ○ Made it much worse

MH4

<u>Over the last two weeks</u>, how often have you been bothered by the following problems?

	Not at all	Several days	More than half the days	Nearly every day
Feeling nervous, anxious or on edge	○	○	○	○
Not being able to stop or control worrying	○	○	○	○
Worrying too much about different things	○	○	○	○
Trouble relaxing	○	○	○	○
Being so restless that it is hard to sit still	○	○	○	○
Becoming easily annoyed or irritable	○	○	○	○
Feeling afraid as if something awful might happen	○	○	○	○
Little interest or pleasure in doing things	○	○	○	○
Feeling down, depressed, or hopeless	○	○	○	○
Trouble falling or staying asleep, or sleeping too much	○	○	○	○
Feeling tired or having little energy	○	○	○	○
Poor appetite or overeating	○	○	○	○
Feeling bad about yourself — or that you are a failure or have let yourself or your family down	○	○	○	○
Trouble concentrating on things, such as reading the [illegible]	○	○	○	○

newspaper or watching television				

Moving or speaking so slowly that other people could have noticed, or so fidgety or restless that you have been moving a lot more than usual	○	○	○	○
Thoughts that you would be better off dead, or thoughts of hurting yourself in some way	○	○	○	○

MH6

Over the past month, how often have you been bothered by the following problems?

	None	A little bit	A moderate amount	A lot
The possibility of you catching COVID-19	○	○	○	○
The possibility of a family member catching COVID-19	○	○	○	○
The possibility of losing your job or other source of income	○	○	○	○
Losing your job due to COVID-19	○	○	○	○
Trying to find a job during COVID-19	○	○	○	○
Working from home	○	○	○	○
The economy	○	○	○	○
Lack of trust in government	○	○	○	○
Concerns about your future	○	○	○	○
Concerns about your loved ones’ (e.g., children’s) future	○	○	○	○
Social isolation/being apart from others	○	○	○	○
Loneliness	○	○	○	○
Conflict with members in your household	○	○	○	○
Your family’s mental health	○	○	○	○
Domestic violence	○	○	○	○
Your use of alcohol and/or other substances	○	○	○	○
[illegible]				

A family member's use of alcohol and/or other substances	○	○	○	○
Childcare	○	○	○	○

MH7

<u>Since the pandemic started in March 2020</u>, have you ever felt you needed help with your mental health?

- ○ Yes
- ○ No

MH8

<u>Since the pandemic started in March 2020</u>, have you received treatment or help for mental health concerns from a medical doctor, psychologist, social worker, psychiatrist, counsellor, support groups, peer counsellor, etc.? (This could be over the phone or in person)

- ○ Yes
- ○ No

MH9

What did you seek treatment or help for? Check all that apply.

- ☐ Depression
- ☐ Anxiety
- ☐ PTSD
- ☐ Bipolar disorder
- ☐ ADHD
- ☐ Stress management
- ☐ Eating disorder
- ☐ Relationship or family problems

- ☐ Other (please specify) ______

MH10

How easy or difficult was it for you to get these services for mental health?

- ○ Very easy
- ○ Easy
- ○ Neither easy nor difficult
- ○ Difficult
- ○ Very difficult

MH11

What made accessing these services difficult? Check all that apply.

- ☐ Long waiting list or wait time for appointment
- ☐ Few or no services available near me
- ☐ No access to internet or computer
- ☐ No transportation
- ☐ Uncomfortable about seeking help
- ☐ Unsure where to get help
- ☐ Costs too much
- ☐ Language or cultural barriers
- ☐ Other (please specify) ______

MH12

Which of the following are reasons you did not get treatment or help for mental health concerns? (Select all that apply)

- ☐ No need for help
- ☐ Didn’t know how or where to get help
- ☐ Prefer to manage problems on my own or by talking to family and friends
- ☐ Didn’t want to seek help by phone or online but couldn’t get help in person
- ☐ Hard to find the time
- ☐ Costs too much
- ☐ Don’t feel comfortable talking about problems
- ☐ Previous bad experiences with seeking help
- ☐ Other (please specify) ________

MH13

Has a medical or psychological professional <u>ever</u> diagnosed you with a mental health disorder (e.g., depression, anxiety, PTSD, bipolar, etc.)?

- ○ Yes
- ○ No

MH14

What were you diagnosed with? Check all that apply.

- ☐ Depression
- ☐ Anxiety
- ☐ PTSD
- ☐ Bipolar disorder
- ☐ ADHD
- ☐ Eating disorder
- ☐ Other (please specify) ________

MH15

When were you diagnosed?

- ○ Before the onset of the pandemic (before March 2020)
- ○ Year 1 of the pandemic (March 2020 – February 2021)
- ○ Year 2 of the pandemic (March 2021 – February 2022)
- ○ Year 3 of the pandemic (March 2022 – current)

INTRO_SU

We are now going to ask about your use of certain substances. We will ask about your consumption now as compared to before the pandemic (March 2020), and we may ask some follow-up questions about more recent usage.

As a reminder, there are no right or wrong answers and your data will never be linked back to your personally.

SU1

In general, how would you describe your alcohol consumption during the pandemic compared to before the pandemic began (March 2020)?

- ○ I do not drink alcohol
- ○ Drank a lot more
- ○ Drank a bit more
- ○ No change
- ○ Drank a bit less
- ○ Drank a lot less
- ○ It varied a lot
- ○ I stopped drinking completely during the pandemic

SU2

How much do you think this change had to do with the pandemic?

- ○ Not at all
- ○ A little
- ○ A lot
- ○ Not sure

SU3

<u>During the past month</u>, how often have you had a drink containing alcohol?

- ○ Never
- ○ Once a month or less
- ○ Two to three times a month
- ○ Once a week
- ○ Two to three times a week
- ○ Four to six times a week
- ○ Daily

SU4

On days when you drink, how many drinks do you usually have? Please provide your best estimate.

[ ]

- ○ N/A
- ○ Refusal
- ○ Do not know

SU5

In general, how would you describe your use of tobacco/nicotine <u>during the pandemic</u> compared to <u>before the pandemic</u> began (March 2020)? This includes cigarettes, e-cigarettes, vaping, chew, pipes or cigars.

- ○ I do not smoke or use tobacco/nicotine
- ○ A lot more
- ○ A bit more
- ○ No change
- ○ A bit less
- ○ A lot less
- ○ It varied a lot
- ○ I quit using tobacco completely during the pandemic

SU6

How much do you think this change had to do with the pandemic?

- ○ Not at all
- ○ A little
- ○ A lot
- ○ Not sure

SU7

<u>During the past month</u>, how often have you smoked or otherwise used tobacco/nicotine?

- ○ Never
- ○ Less than once a week, but at least once a month
- ○ Less than daily, but at least once a week

○ Daily

SU8

How do you most often use tobacco/nicotine? Please choose one.

○ Cigarettes
○ E-cigarettes
○ Vaping/vaporizers
○ Chew
○ Cigars or pipe-smoking
○ Other (please specify) ____________

SU8A

<u>During the past week</u>, about how many cigarettes did you smoke each day?

○ 1 to 5 (less than a quarter pack)
○ 6 to 10 (between one quarter and a half pack)
○ 11 to 15 (between half a pack and three-quarters pack)
○ 16 to 20 (between three-quarters pack and a full pack)
○ More than 20 (more than a full pack)

SU9

In general, how would you describe your cannabis consumption (excluding prescription cannabis) <u>during the pandemic</u> compared to <u>before the pandemic</u> began (March 2020)?

By cannabis, we mean any cannabis product (including THC and CBD), including dried cannabis, liquid or solid extracts, edibles or drinks, oil sprays, capsules, etc.

- ○ I do not use cannabis
- ○ A lot more
- ○ A bit more
- ○ No change
- ○ A bit less
- ○ A lot less
- ○ It varied a lot
- ○ I stopped using cannabis completely during the pandemic

SU10

How much do you think this change had to do with the pandemic?

- ○ Not at all
- ○ A little
- ○ A lot
- ○ Not sure

SU11

<u>During the past month</u>, how often have you used any form of cannabis (excluding prescription cannabis)?

- ○ Never
- ○ Once a month or less
- ○ Two to three times a month
- ○ Once a week
- ○ Two to three times a week
- ○ Four to six times a week
- ○ Daily

SU12

How do you use cannabis? (Check all the apply)

- ☐ Smoking
- ☐ Eating or drinking
- ☐ Vaporizing (using an e-cigarette, vape, vaporizer)
- ☐ Oils or sprays for oral use
- ☐ Capsules
- ☐ Dabbing
- ☐ Other (please specify) ______

SU13

Have you been prescribed any of the following types of drugs by a medical professional since March 2020? Please check all that apply.

- ☐ Opioids (painkillers, e.g., codeine, oxycodone, hydromorphone, morphine; methadone, etc.)
- ☐ Sedatives (Benzodiazepines; e.g. Ativan, Xanax, Valium, Dalmane, etc.)
- ☐ Non-benzodiazepine sedatives (e.g., for insomnia; Ambien, Imovane, Zopiclone) or barbiturates (e.g., Phenobarbital, Amytal, Luminal))
- ☐ Stimulants (e.g., Adderall, Concerta, Ritalin, Dexedrine, etc.)
- ☐ Cannabis
- ☐ None of the above

SU14

Compared to before the pandemic began (March 2020), has the amount or how often you take this drug changed?

	Increased	Decreased	Remained the same
Opioids	○	○	○
Sedatives (benzodiazepines)	○	○	○
Sedatives (non-benzodiazepines) and barbiturates	○	○	○
Stimulants	○	○	○
Cannabis	○	○	○

SU15

How much do you think these changes had to do with the pandemic?

- ○ Not at all
- ○ A little
- ○ A lot
- ○ Not sure

SU16

How would you describe your *other drug use* now compared to before the pandemic began (March 2020)? By other drug use, we mean drugs such as: cocaine, fentanyl, methamphetamine, ecstasy, magic mushrooms/psylocybin, LSD, acid, inhalants, etc.; or prescription drugs that are not your own or that have been altered for use.

- ○ I do not use any of these other drugs
- ○ A lot more
- ○ A bit more
- ○ No change
- ○ A bit less
- ○ A lot less
- ○ It varied a lot
- ○ I stopped using completely during the pandemic

SU17

How much do you think this change had to do with the pandemic?

- ○ Not at all
- ○ A little
- ○ A lot

○ A lot

○ Not sure

SU18

<u>During the past month</u>, how often have you used other drugs such as: cocaine, fentanyl, methamphetamine, ecstasy, hallucinogenics, inhalants, etc.; or prescription drugs that are not your own or that have been altered for use?

○ Never

○ Once a month or less

○ Two to three times a month

○ Once a week

○ Two to three times a week

○ Four to six times a week

○ Daily

SU19

Which other drugs do you most commonly use? Please list all types. (e.g. cocaine, fentanyl, methamphetamine, ecstasy, etc.)

○ N/A

○ Refusal

○ Do not know

SU20

<u>Since the pandemic began (March 2020)</u>, how concerned have you been about your use of the following?

	Not at all	Slightly	Somewhat	A lot	Extremely
Alcohol	○	○	○	○	○
Tobacco	○	○	○	○	○
Cannabis	○	○	○	○	○
Prescribed drugs	○	○	○	○	○
Other drugs	○	○	○	○	○

SU21

<u>Since the pandemic began (March 2020)</u>, have you accessed services to address your substance use concerns (e.g. inpatient or outpatient treatment, a medical doctor, psychologist or psychiatrist, social worker, counsellor, harm reduction workers, AA/NA or other support groups, peer supports, etc.)? This could be on the internet, phone or in person.

- ○ Yes
- ○ No

SU23

How easy or difficult was it for you to get these services?

- ○ Very easy
- ○ Easy
- ○ Neither easy nor difficult
- ○ Difficult
- ○ Very difficult

SU24

What made accessing these services difficult? Check all that apply.

- ☐ Long waiting list or wait time for appointment
- ☐ Few or no services available near me
- ☐ No access to internet or computer
- ☐ No transportation
- ☐ Uncomfortable about seeking help
- ☐ Unsure where to get help
- ☐ Costs too much
- ☐ Language or cultural barriers
- ☐ Other (please specify)

SU25

Which of the following are reasons you did not seek services to address your substance use concerns? (Select all that apply)

- ☐ Did not feel the need to access services
- ☐ Did not know where to go or how to access help
- ☐ Don't want to seek help via phone or online but can't get help in person
- ☐ Hard to find the time
- ☐ Costs too much
- ☐ Don't feel comfortable talking about problems
- ☐ Previous bad experiences with seeking help
- ☐ Prefer to manage problems on my own or by talking to family and friends

- ☐ Other (please specify) ______

SU26

Has a medical or psychological professional ever diagnosed you with a substance use disorder (e.g., alcohol use disorder, cannabis use disorder, opioid use disorder, stimulant use disorder, other substance use disorder, etc.)?

- ○ Yes
- ○ No

SU27

What were you diagnosed with? Check all that apply.

- ☐ Alcohol use disorder
- ☐ Cannabis use disorder
- ☐ Opioid use disorder
- ☐ Stimulant use disorder
- ☐ Other substances use disorder

SU28

When were you diagnosed?

- ○ Before the onset of the pandemic (before March 2020)
- ○ Year 1 of the pandemic (March 2020 – February 2021)
- ○ Year 2 of the pandemic (March 2021 – February 2022)
- ○ Year 3 of the pandemic (March 2022 – current)

D1

In what year were you born?

[ ]

- ○ N/A
- ○ Refusal
- ○ Do not know

D2

What is your gender identity?

- ○ Woman
- ○ Man
- ○ Male to female transgender (MTF)
- ○ Female to male transgender (FTM)
- ○ Non-binary
- ○ Two-spirit
- ○ Prefer to self-identify as: [ ]

D3

Please select the group(s) you identify with from the list below:

Select all that apply.

- ☐ White or Caucasian
- ☐ First Nations
- ☐ Métis
- ☐ Inuk (Inuit)
- ☐ South Asian (e.g., East Indian, Pakistani, Sri Lankan)
- ☐ Chinese
- ☐ Black or African Canadian
- ☐ Filipino
- ☐ Latin American
- ☐ Arab
- ☐ Southeast Asian (e.g., Vietnamese, Cambodian, Malaysian, Laotian)
- ☐ West Asian (e.g., Iranian, Afghan)
- ☐ Korean
- ☐ Japanese
- ☐ Other (Please specify) ____________

D6

Do you identify yourself as a person of color?

- ○ Yes
- ○ No

D4

What is the highest level of education that you have?

- ○ Less than high school
- ○ High school diploma or equivalent
- ○ Skilled trades training
- ○ Some college or university
- ○ College or university degree or higher
- ○ Other (please specify) [ ]

D5

Did you move to Canada in the past 5 years?

- ○ Yes
- ○ No

D7

Do you identify as any of the following? Check all that apply.

- ☐ An individual with a physical disability
- ☐ An individual with chronic pain
- ☐ I do not identify as either of these

D8

What is your six-digit postal code? (no spaces, e.g., S7N5A5)

- N/A
- Refusal
- Do not know

COMMENTS

If you would like to add any additional comments about your experiences during the pandemic, please write them here. Then click "Next".

- N/A
- Refusal
- Do not know
