# Supplementary material for: Capturing and Documenting the Wider Health Impacts of the COVID-19 Pandemic Through the Remember Rebuild Saskatchewan Initiative: Protocol for a Mixed Methods Interdisciplinary Project
Source: JMIR Res Protoc. 2023 Jun 6;12:e46643. doi: 10.2196/46643 (PMC10282902; doi:10.2196/46643)
Supplement: Multimedia Appendix 2 [file resprot_v12i1e46643_app2.pdf]

Muhajarine N, Dixon J, Dyck E, Clifford J, Chassé P, Gupta SD, Christopherson-Cote C, Remember Rebuild Sask atchewan Team. Capturing and Documenting the Wider Health Impacts of the COVID-19 Pandemic Through th e Remember Rebuild SaskatchewanInitiative: Protocol for a Mixed Methods Interdisciplinary Project JMIR Res Protoc 2023;12:e46643. URL: https://www.researchprotocols.org/2023/1/e46643/ doi: 10.2196/46643.

**Appendix 2**
**Build Back Better Survey**
**Service Provider Interview Guide**

**Interview script:**

Thank you so much for meeting with me today. As you know from our review of the consent form, we are seeking your perspective on the COVID-19 pandemic in Saskatchewan. We would like to hear how your ability to deliver services has been affected since the pandemic began in March 2020. We are going to ask about your thoughts and experiences as a person delivering services in one (or more) areas including housing, food, mental health, and substance use (such as harm reduction, treatment, or recovery).

While we have a few guiding questions for this study, if you want to include additional information or if you think we have missed something, please feel free to include that too.

I'll start by asking a few questions to get us started. The interview should take no more than 60 minutes. If you would like to skip a question, take a break, or end the interview at any time please just let me know. I will be recording the interview, but you may ask me to stop at any time.

You are welcome to say as much, or as little, as you like about any particular question as you like. There are no wrong answers and no pressure or obligation to continue. Do you have any questions or concerns before we get started?

Now we can get into the more formal study questions and please feel free to say 'that doesn't make sense' or 'what does that mean' if anything is not clear.

**Questions:**

1) Tell me about the first time you heard about COVID-19 in your town / city / etc. [in Saskatchewan]?

2) What services did you offer before the pandemic?

3) Reflecting on the services you provided since March 2020, the beginning of the pandemic?

    a. How did demand for your services change during the pandemic? (Increase, Stay the Same, or Decrease)
    b. Did (more / fewer / the same) number of people come seeking services during the pandemic? Who were the people (ie older / younger than before, etc.)
    c. Did you develop new services to meet service user needs?

4) Has COVID-19 increased pressure on you to offer more services to help with food / housing / mental health or substance use (such as harm reduction, treatment, or recovery)?
    a. When did you first notice changing demand?
    b. Did you continue to feel this as the pandemic continued?
    c. Can you tell me more about this change in service demand and what you think caused it?

5) What were the overall impacts of the pandemic on the services [NAME CBO] offered? How did the pandemic impact the way services were accessed?
    **a. Housing**
        i. How did the pandemic affect housing needs in the community your organization serves?
        ii. How did the pandemic affect housing supply?
    **b. Food Security**
        i. How did the pandemic affect demand for food in the community your organization serves?
        ii. How did the pandemic affect supply and access to food?
    **c. Mental Health**
        **i.** How did the pandemic affect mental health needs in the community your organization serves?
        **ii.** How did the pandemic affect access to mental health services?
    **d. Substance Use**
        **i.** How did the pandemic affect substance use in the community your organization serves?
        **ii.** How did the pandemic affect access to harm reduction, treatment, or recovery services?

6) How did the pandemic change the way you delivered services?
    a. Did you expand or reduce services?
    b. Did you offer clients access to remote service, via telephone or other digital resources?
    c. What barriers did you encounter when offering in person services? (financial, resources, personnel, access and use of PPE, pandemic restrictions, etc.)

7) During the pandemic, where did you turn for financial or logistical support? (ie Federal, Province, Municipality, or NGO?)
    a. Did you receive the aid you requested? What barriers did you face in receiving assistance so you could provide services?

8) Do you feel like the pandemic is still affecting your ability to deliver services? How has demand changed since March 2022, when the province removed pandemic restrictions like masks, vaccinations, etc.?

9) Are you comfortable sharing with me if you have you ever had COVID-19? Did you have it multiple times? Are you fully recovered now? Are you still experiencing symptoms?
    a. Did it affect you or your organization's ability to provide services?

10) What is the most important thing for people to know about when providing services to help with access to housing, food, mental health, or substance use services (such as harm reduction, treatment, or recovery)?

11) What do you think would makes accessing food, housing, mental health or substance use (such as harm reduction, treatment, or recovery) services easier in your community? What makes it harder?

12) Is your organization willing to share any data that you collected on service use volumes during the pandemic? If we report on these numbers in future research outputs will anonymize the source so your agency is not identified.

13) What else would you like to share about your experiences during the pandemic?

14) How do you think we should share our research results so that it can reach more people?

15) [For service providers in core focus areas] We are also interested in getting perspectives from people who access services for this work. Would you be willing to help us connect with someone who accesses services from your organization for an interview?
    a. Is a $25 honorarium reasonable compensation for someone accessing services?
    b. Do you have other recommendations for us to consider in our recruitment of people accessing services?
    c. Can we follow up with you to discuss setting up an interview with someone accessing services from your agency?

16) Are you interested in attending a follow-up discussion where we share results and provide opportunities for feedback on whether the findings make sense? Options will be available so you can participate without revealing personal information or your identity to protect your confidentiality.
        i. If yes, how would you prefer to be notified about the discussion forum? Email or telephone?
