# Supplementary material for: Capturing and Documenting the Wider Health Impacts of the COVID-19 Pandemic Through the Remember Rebuild Saskatchewan Initiative: Protocol for a Mixed Methods Interdisciplinary Project
Source: JMIR Res Protoc. 2023 Jun 6;12:e46643. doi: 10.2196/46643 (PMC10282902; doi:10.2196/46643)
Supplement: Multimedia Appendix 3 [file resprot_v12i1e46643_app3.pdf]

Muhajarine N, Dixon J, Dyck E, Clifford J, Chassé P, Gupta SD, Christopherson-Cote C, Remember Rebuild Saskatchewan Team. Capturing and Documenting the Wider Health Impacts of the COVID-19 Pandemic Through the Remember Rebuild SaskatchewanInitiative: Protocol for a Mixed Methods Interdisciplinary Project JMIR Res Protoc 2023;12:e46643. URL: https://www.researchprotocols.org/2023/1/e46643/
doi: 10.2196/46643.

Appendix 3
Build Back Better Survey
People Accessing Services Interview Guide

Interview script:

Thank you so much for meeting with me today. As you know from [NAME CBO partner] and our review of the consent form, we are seeking your perspective on the COVID-19 pandemic in Saskatchewan. We would like to hear how your ability to access services has been affected since the pandemic began in March 2020. We are going to ask about your thoughts and experiences as a person accessing services in one (or more) areas including housing, food, mental health, and substance use.

While we have a few guiding questions for this study, if you want to include additional information or if you think we have missed something, please feel free to include that.

I'll start by asking a few questions to get us started. The interview should take no more than 30-40 minutes. You are welcome to say as much, or as little, as you like about any question as you like. There are no wrong answers! If you would like to skip a question, take a break, or end the interview at any time please just let me know. I will be recording the interview, but you may ask me to stop at any time.

Your $25 honorarium for participation in the study will be provided to you now, so there is no pressure or obligation to continue. Do you have any questions or concerns before we get started?

Now we will start the questions. Please feel free to say 'that doesn't make sense' or 'what does that mean' if anything is not clear.

Questions:

1. Can you tell me about the first time you heard about COVID-19 in your town / city / etc. [in Saskatchewan]? Where were you? How did you feel?

2. Were you connected to [service providing agency] in March 2020 when the pandemic shutdown started?
    a. Can you share with me which services you have been using at (service providing agency) or who you have been connecting with here?
    b. Are there any other organizations or services elsewhere that you visit?

Muhajarine N, Dixon J, Dyck E, Clifford J, Chassé P, Gupta SD, Christopherson-Cote C, Remember Rebuild Saskatchewan Team. Capturing and Documenting the Wider Health Impacts of the COVID-19 Pandemic Through the Remember Rebuild SaskatchewanInitiative: Protocol for a Mixed Methods Interdisciplinary Project JMIR Res Protoc 2023;12:e46643. URL: https://www.researchprotocols.org/2023/1/e46643/
doi: 10.2196/46643.

3. After the shutdown, were you able to visit these organizations for their services?
    a. If yes:
        i. Were they easier, harder, or about the same to access? Why?
        ii. Did you use their services differently compared to before the pandemic? E.g. virtual, telephone, remote delivery? How was this?
        iii. How did you feel about following public health guidelines (like masking, distancing, vaccinations) when visiting?
    b. If no:
        i. Did not being able to visit these services impact you? How?
        ii. Did you find somewhere else to go? Where?

4. Besides the organizations you mentioned, did you turn to anyone else for support or help during the pandemic?
    a. Financial, material [food, housing, goods or supplies], emotional supports?
    b. Other organizations, government, family, friends, community groups, religious groups?

5. Can you share with me what you needed help/support with the most during the pandemic (financial, material, emotional, other?)?
    a. Did you ever need help with your housing? E.g. finding a place to stay, getting evicted or kicked out of housing, landlord issues, etc.? Was this due to the pandemic?
    b. Did you ever need help with food? E.g. having adequate food for yourself and/or your family, getting meals or groceries, preparing meals, etc.? Was this due to the pandemic?
    c. Did you ever need help with your mental health? E.g. talking to someone, feeling stressed or anxious, challenges dealing with emotions, etc.? Was this due to the pandemic?
    d. Did you ever need help dealing with substance use? E.g. alcohol, tobacco, cannabis or other substances, accessing treatment or harm reduction services, using substances safely, etc.? Was this due to the pandemic?

6. Of the organizations you visited, were there any that you think did a really good job offering services during the pandemic? Why? What did they do?

Muhajarine N, Dixon J, Dyck E, Clifford J, Chassé P, Gupta SD, Christopherson-Cote C, Remember Rebuild Saskatchewan Team. Capturing and Documenting the Wider Health Impacts of the COVID-19 Pandemic Through the Remember Rebuild SaskatchewanInitiative: Protocol for a Mixed Methods Interdisciplinary Project JMIR Res Protoc 2023;12:e46643. URL: https://www.researchprotocols.org/2023/1/e46643/
doi: 10.2196/46643.

7. Of the organizations you visited, was there anyone that you think could have done a better job offering services during the pandemic? Why? What did they do/not do?

8. Is there anything you think is important for service providers or leaders in this work to know in offering these types of services?

9. Were there ever times during the pandemic when you needed help or support with something but didn't have anyone to ask?
    a. What did you need at that time?
    b. What did you do about it?
    c. How often did this happen?

10. Did you ever get COVID-19? Can you tell me more about that?
    a. Were you able to isolate? How and where?
    b. Did any person or organization help you while you had it?

11. Now that it's been over 3 years since the pandemic was declared globally and public health restrictions have been lifted, do you think we're back to normal?
    a. Is the pandemic still affecting the organizations or services you visit? How?
    b. Is the pandemic still affecting you? How?

12. Is there anything else you'd like to share about your experiences during the pandemic?

13. Would you like to see and give feedback on findings from this work?
    a. Who else do you think should see our findings?
