# Supplementary material for: Capturing and Documenting the Wider Health Impacts of the COVID-19 Pandemic Through the Remember Rebuild Saskatchewan Initiative: Protocol for a Mixed Methods Interdisciplinary Project
Source: JMIR Res Protoc. 2023 Jun 6;12:e46643. doi: 10.2196/46643 (PMC10282902; doi:10.2196/46643)
Supplement: Multimedia Appendix 4 [file resprot_v12i1e46643_app4.pdf]

Canadian Institutes of Health Research
160 Elgin Street, 9th Floor
Address Locator 4809A
Ottawa, Ontario K1A 0W9

Instituts de recherche en santé du Canada
160, rue Elgin, 9e étage
Indice de l'adresse 4809A
Ottawa (Ontario) K1A 0W9

Institute of Aging
Institute of Cancer Research
Institute of Circulatory and Respiratory Health
Institute of Gender and Health
Institute of Genetics
Institute of Health Services and Policy Research
Institute of Human Development and Child and Youth Health
Institute of Indigenous Peoples' Health
Institute of Infection and Immunity
Institute of Musculoskeletal Health and Arthritis
Institute of Neurosciences, Mental Health and Addiction
Institute of Nutrition, Metabolism and Diabetes
Institute of Population and Public Health

Institut du vieillissement
Institut du cancer
Institut de la santé circulatoire et respiratoire
Institut de la santé des femmes et des hommes
Institut de génétique
Institut des services et des politiques de la santé
Institut du développement et de la santé des enfants et des adolescents
Institut de la santé des Autochtones
Institut des maladies infectieuses et immunitaires
Institut de l'appareil locomoteur et de l'arthrite
Institut des neurosciences, de la santé mentale et des toxicomanies
Institut de la nutrition, du métabolisme et du diabète
Institut de la santé publique et des populations

February 28, 2022

Dr. Nazeem Muhajarine
Department of Community Health and Epidemiology
College of Medicine
University of Saskatchewan
104 Clinic Place
Saskatoon, Saskatchewan S7N 5E5

Dear Dr. Muhajarine,

On behalf of the Government of Canada, the Canadian Institutes of Health Research (CIHR) is pleased to inform you that your recent application "Data and equity needed to drive post-pandemic recovery in Canada: a Saskatchewan contribution", submitted to the Operating Grant: Addressing the Wider Health Impacts of COVID-19, has been approved for funding.

As a successful nominated principal applicant (NPA), please note that there are additional Conditions of Funding outside of CIHR's standard requirements related to this competition (please refer to the Conditions of Funding section of the funding opportunity and your Authorization for Funding form (AFF) for more details). Furthermore, if you are in receipt or become eligible to receive any funding from another source for any part of this project, you must advise CIHR immediately by following the instructions outlined in the "Funding Overlap Declaration" form (https://cihr-irsc.gc.ca/e/797.html). Failure to self-declare overlap could lead to CIHR cancelling all funding related to this grant.

The decisions of this funding opportunity are under embargo. CIHR Communications will be in touch with you in the near future regarding plans for an official public announcement of the decisions. We would ask grant recipients and their institutions to hold off on any public communications (e.g., news releases, external web postings, social media) until the official announcement has taken place.

As outlined in CIHR's equity strategy (https://cihr-irsc.gc.ca/e/50068.html), CIHR is committed to creating an equitable funding system by identifying and eliminating systematic biases towards any individual or group that would hinder access to CIHR funds. CIHR committed to ensuring a minimum proportion of funding was allocated to applications related to Indigenous Health Research (IHR) and that the proportion of grants awarded to applications submitted in French and applications submitted by female nominated principal applicants was not less than the proportion of applications submitted by those groups. However, for this competition, the proportion of applications for all groups was met without the need to apply the equalization process.

As CIHR does not notify co-applicants of the decision, we ask that you inform those individuals involved, along with their research institutions (if different from your own), of the outcome of this application.

Please note that NPIs may be contacted by CIHR to complete a short survey regarding barriers and enablers to their research. CIHR is committed to promoting opportunities for knowledge sharing and collaboration to facilitate and accelerate Canada's research response to the COVID-19 outbreak. As such, the results of the survey may be used to support future activities related to knowledge mobilization in the COVID-19 context.

CIHR would like to remind grant recipients that all COVID-19 related publications must be open access, in alignment with the call from the Chief Science Advisors (https://www.ic.gc.ca/eic/site/063.nsf/eng/h_98016.html), and that all data produced as a result of this funding must be shared in line with the Joint statement on sharing research data and findings relevant to the novel coronavirus (nCoV) outbreak (https://wellcome.org/coronavirus-covid-19/open-data). Data related

Canada
CIHR IRSC
Discoveries for life / Découvertes pour la vie

to research by and with First Nations, Métis, or Inuit communities whose traditional and ancestral territories are in Canada must be managed in accordance with data management principles developed and approved by those communities, and on the basis of free, prior and informed consent. This includes, but is not limited to, considerations of Indigenous data sovereignty, as well as data collection, ownership, protection, use, and sharing.

Should you require additional information, please contact CIHR at EHTRF-FRNMS@cihr-irsc.gc.ca.

Sincerely,

Tammy Clifford, PhD
Vice-President, Research Programs
Research Programs Portfolio

Review Type/Type d'évaluation: Committee Member 1/Membre de comité 1
Name of Applicant/Nom du chercheur: Muhajarine, Nazeem
Application No./Numéro de demande: 475084
Agency/Agence: CIHR/IRSC
Competition/Concours: 2021-11-08 Operating Grant: Addressing the Wider Health Impacts of COVID-19/Subvention de fonctionnement : Étude des répercussions globales de la COVID 19 sur la santé
Committee/Comité: Operating Grant : Addressing the Wider Health Impacts of COVID-/Sub. de fonct.:Étude des répercussions globales de la COVID 19 sur la santé
Title/Titre: Data and equity needed to drive post-pandemic recovery in Canada: a Saskatchewan contribution

---

Assessment/Évaluation:

Proposed Research:
- The prosposed project plans to conduct a repeated cross-sectional survey (4 time points in 2022) to assess the prevalence of food insecurity, housing precarity, and mental health and substance use issues in Saskatchewan.
- The proposed project responds to all three objectives of the funding call, although it's relevance to objective #2 (identify effective interventions) could have been more clearly addressed.
- In addition to the repeated surveys, the investigators are proposing to pull data on the pre-pandemic and earlier pandemic periods from a range of sources, including service-related data from providers, as well as other population-based surveys done in the province (e.g. CCHS, MHCC surveys, etc.). This portion of the project is not well described at all, despite being fundamental to inferring any changes over time due to the pandemic. It is not clear whether the investigators will be accessing primary data from these providers and surveys, building a data repository, or pulling aggregate data on key indicators. Additionally, comparability with the measures included in the primary data collection is not mentioned (e.g. will substance use be measured using the same tool?), and there may be fundamental differences in the populations included across the data sources. WIthout this fundamental information, it is unclear how the investigators plan to assess changes over time, and whether they will be able to attribute any observed changes to pandemic related events (vs. differences in measurement tools, samples, etc.).
- The inclusion of service provider and policy maker perspectives through the Delphi approach is a notable strength, and an important iKT strategy for this project.

Impact of the Research:
- The investigators have pulled together an impressive list of community partners and knowledge users, which is a notable strength of this proposal and increases the likelihood of impact.
- Without a clearer understanding of what data will be obtained and how it will be used, it's difficult to assess the potential impact. I can envision that the collation and monitoring of key metrics would be very useful to local decision-makers (e.g. an indicator dashboard broken down by health region), but this hasn't been clearly articulated in the current proposal.
- It is also difficult to assess the feasibility, and potential for timely results - for example, the investigators mention obtaining data from 120 community based organizations across SK, which could be very time consuming and costly if platforms and partnerships are not already established.
- The inclusion of the study data in Saskatchewan's COVID-19 public archive is novel and a noteworthy strength.

Applicants
- The team includes investigators from at a range of different career stages, and trainees are well integrated into the research plan.
- The plan to incorporate EDI is excellent, well done.

**Review Type/Type d'évaluation:**	Committee Member 1/Membre de comité 1
**Name of Applicant/Nom du chercheur:**	Muhajarine, Nazeem
**Application No./Numéro de demande:**	475084
**Agency/Agence:**	CIHR/IRSC
**Competition/Concours:**	2021-11-08 Operating Grant: Addressing the Wider Health Impacts of COVID-19/Subvention de fonctionnement : Étude des répercussions globales de la COVID 19 sur la santé
**Committee/Comité:**	Operating Grant : Addressing the Wider Health Impacts of COVID-/Sub. de fonct.:Étude des répercussions globales de la COVID 19 sur la santé
**Title/Titre:**	Data and equity needed to drive post-pandemic recovery in Canada: a Saskatchewan contribution

Assessment/Évaluation:

Budget:
- The quote for surveys estimates a 10 minute survey per person, which seems very short given the extensive amount of data the investigators are proposing to collect.

Review Type/Type d'évaluation: Committee Member 2/Membre de comité 2
Name of Applicant/Nom du chercheur: Muhajarine, Nazeem
Application No./Numéro de demande: 475084
Agency/Agence: CIHR/IRSC
Competition/Concours: 2021-11-08 Operating Grant: Addressing the Wider Health Impacts of COVID-19/Subvention de fonctionnement : Étude des répercussions globales de la COVID 19 sur la santé
Committee/Comité: Operating Grant : Addressing the Wider Health Impacts of COVID-/Sub. de fonct.:Étude des répercussions globales de la COVID 19 sur la santé
Title/Titre: Data and equity needed to drive post-pandemic recovery in Canada: a Saskatchewan contribution

Assessment/Évaluation:

Muhajarine Nazeem / Erika Dyck, PA

Data and equity needed to drive post-pandemic recovery in Canada: a Saskatchewan contribution
The present is a multi-methods, 2 years cohort study. The project has four research questions. The first two address prevalence of adverse mental health status, substance use, home evictions and housing precarity, and food insecurity in Saskatchewan, as well as analysis by equity seeking groups (Indigenous groups, visible minority/newcomers, LGBTQ2S+, people with disabilities) and those with the greatest social and economic needs (unemployed or under-employed, low-income), in these four areas. Quantitative data will be mostly gathered by telephone survey (approx.10 min) although other groups with less accessibility will be reached through community partners. Qualitative data will be collected using Delphi and World Café approach to capture experiences from services providers that will be used to address the last two research questions: What is the role of front line human services played in helping during the pandemic, what challenges encountered and what make services effective? What do service users and providers believe are priorities for policy and practice in these areas?

Strengths:
The project presents a compelling topic and has a strong team. It is clear that builds on community partners and on prior projects. Before the pandemic, significant numbers of Saskatchewan residents experienced difficulties related to the intersecting issues the team has experience working with (e.g., housing, food insecurity). The team has expertise and ties in the community that would likely leverage knowledge translation effectively. Regarding the specific components, the housing precarity and food insecurity are thoughtfully delineated and clearly specified. The gap from the evidence to what this study can add is explicit, and aligned in the measurements, analysis and support letters. Another strength of the proposal is the plan for how Equity, Diversity and Inclusion (EDI) and Indigenous Rights (IR) will be incorporated into the research proposed. The study is lead by a BIPOC woman and the team is diverse. This will support meaning engagement with these groups, as they are represented as participants in the research and investigational team. Finally, there is a lack of evidence from Saskatchewan in the areas identified by the team, and this study can provide a first step of evidence in a feasible and meaningful way, due to the methodology selected (multi-methods). It addresses the objectives of the funding opportunity by understanding the impact of the pandemic on mental health status, substance use, home evictions and housing precarity, and food insecurity in Saskatchewan among priority groups and by looking to identify service and policy priorities through engaging with community-based service agencies, including data gathered from front-line service providers and program deliverers, and formulating recommendations for government policies

Areas less developed: The telephone survey will be complemented with other methods, such as word of mouth, leaning on community partners, to reach people that have not access to phones or residencies where they can be reached. These groups are key for equity seeking, and the four areas identified, as they might represent the lower bound in scores. However, there is no explanation how they will be interviewed, privacy, payment for equity, etc. It is specified that 200 people will be in this data collection method groups, and likely this will be the one that will take up most of the resources. There is concern on how this will be done and how this will affect the research questions 1 and 2. The measurements in the Substance Use area are underdeveloped. Asking questions that will yield a yes or no answer in the prior months will not capture this dimension. This section requires more

**Review Type/Type d'évaluation:**	Committee Member 2/Membre de comité 2
**Name of Applicant/Nom du chercheur:**	Muhajarine, Nazeem
**Application No./Numéro de demande:**	475084
**Agency/Agence:**	CIHR/IRSC
**Competition/Concours:**	2021-11-08 Operating Grant: Addressing the Wider Health Impacts of COVID-19/Subvention de fonctionnement : Étude des répercussions globales de la COVID 19 sur la santé
**Committee/Comité:**	Operating Grant : Addressing the Wider Health Impacts of COVID-/Sub. de fonct.:Étude des répercussions globales de la COVID 19 sur la santé
**Title/Titre:**	Data and equity needed to drive post-pandemic recovery in Canada: a Saskatchewan contribution

Assessment/Évaluation:

focus and thinking on what about substance use is relevant for this particular project and how it will be used. There are too many groups and they will be using them quite differently. There will be sample size issues for certain drugs, while others without detailed questions will not give you a full picture by, for example, gender (e.g., use of alcohol differs by age and gender, but has to be asked beyond yes/no). There is a similar issue with the mental health variables. There is a lack of focus on what will be collected and analyzed and how different groups answer or access services from where data will be extrapolated for mental health.
Analytical strategy: For research question 1 and 2 (quantitative), the analysis seem sound, although quite generic. The analysis indicates “outcome data”, however… what is the outcome? There are several measures and it is not totally clear how the main outcomes are identified particularly for MH (mental health) and SU (substance use). If for SU is a yes/no use as described, there will not be enough sensitivity to make meaningful conclusions. For example, when bars closed people started drinking at home more. This could have yielded an increase in alcohol use just for the price change or all the opposite, bars closed, less drinking. These connections are complex, interactions are relevant, since lower alcohol could have led to increase in other substances. There is a generic list of analysis that is not clear what is the main outcome and what connections are expected, in particular for the equity seeking groups. The MH variables are treated quite general too. Is the objective to look at an overall score or a specific trait. Depending on the answer, sample size will matter. Is there enough sample to have representation by diagnosis? This lack of representation by diagnosis can lead to potential biases or underreporting, specially when people might have never accessed services where to get diagnosed.
For research question 3 and 4 (qualitative), while this objective is a strength of the proposal, there is very little regarding analytical strategy beyond that will be a thematic analysis. Also, it is not described how representativeness will be preserved and compensation for participation for equity seeking groups.

The remembering and rebuilding project is brought at the end, with little explanation with the connection and how is the information collected in this project being “layered”.

Other comments:

The project responds adequately to SGBA:There are analysis planned by gender in research question 2. Also, while not identified how this will be done, the investigators indicate the project is focused primarily on gender, on social dimensions of COVID-19 in Saskatchewan (impact, short-term and potentially longer-term) and its people, including but not limited to roles, power dynamics and trait societies ascribe to women and men.

Budget: 2 Years requested, $229,091. Overall no comments on the budget, seems adequate. Compensation to people with live/d experiences when contributing with their time to research is not clearly indicated.

EDI: An excellent EDI statement. A groups with true representation and constant work in progress.

Review Type/Type d'évaluation: Committee Member 3/Membre de comité 3
Name of Applicant/Nom du chercheur: Muhajarine, Nazeem
Application No./Numéro de demande: 475084
Agency/Agence: CIHR/IRSC
Competition/Concours: 2021-11-08 Operating Grant: Addressing the Wider Health Impacts of COVID-19/Subvention de fonctionnement : Étude des répercussions globales de la COVID 19 sur la santé
Committee/Comité: Operating Grant : Addressing the Wider Health Impacts of COVID-/Sub. de fonct.:Étude des répercussions globales de la COVID 19 sur la santé
Title/Titre: Data and equity needed to drive post-pandemic recovery in Canada: a Saskatchewan contribution

---

Assessment/Évaluation:

A. Evaluation Criteria

1. Proposed Research

The application describes a project aimed at generating knowledge that will permit Saskatchewan to 'build back better' following the COVID-19 pandemic. This study, co-led by an epidemiologist and historian of medicine, will generate data reflecting the impact of the pandemic on mental health, substance use, housing precarity, and food insecurity among residents of Saskatchewan. While there is Saskatchewan-specific data on mental health and substance use problems during the pandemic, less is known about changes in food insecurity and housing precarity and evictions in Saskatchewan during the pandemic. This project aims to provide this prevalence data. This is one of the notable strengths of this application. This project will also use the prevalence data to offer direction for policy and service changes and, another notable strength, this project will examine how the pandemic has differentially impacted those in equity-seeking groups. The project addresses all three primary aims of this funding call.

Strengths

This is a well written proposal. The study design and analytic plan map onto the research questions. Issues of diversity are considered throughout the proposal.

Weaknesses

The fit of this project with objective 2 of this funding call is a bit less obvious (than fit with the other objectives). More detailed/specific KMb plan related to this objective would have been helpful.

Data will be gathered from front-line service providers and program deliverers to supplement participant data (as part of RQ1 & RQ2). The related methods and procedures are unclear. What measures will be included? How will these data be analyzed and used in conjunction with participant data?

Procedures for recruitment to address RQ3 & RQ4 are unclear. How will data from service users be included here? Is this from data collected in multi-wave study?

Goals related to understanding services used and barriers to service use during the pandemic seem underdeveloped. Will this information be used to interpret prevalence data? This leads to my one lingering concern: there is a lot of data to be collected, which is a strength, but I wonder how it will be integrated to develop directions for intervention. For example, will service use and satisfaction be tested as potential protective factors?

**Review Type/Type d'évaluation:**	Committee Member 3/Membre de comité 3
**Name of Applicant/Nom du chercheur:**	Muhajarine, Nazeem
**Application No./Numéro de demande:**	475084
**Agency/Agence:**	CIHR/IRSC
**Competition/Concours:**	2021-11-08 Operating Grant: Addressing the Wider Health Impacts of COVID-19/Subvention de fonctionnement : Étude des répercussions globales de la COVID 19 sur la santé
**Committee/Comité:**	Operating Grant : Addressing the Wider Health Impacts of COVID-/Sub. de fonct.:Étude des répercussions globales de la COVID 19 sur la santé
**Title/Titre:**	Data and equity needed to drive post-pandemic recovery in Canada: a Saskatchewan contribution

Assessment/Évaluation:

It is noted that "about three times fewer in-person consultations with mental health professionals have been reported". This seems to ignore the increase in use of telehealth. Will use of teletherapy be considered?

2. Impact of the Research

Importance of project is clearly justified. This project has the potential to generate prevalence data specific to Saskatchewan on food insecurity and housing precarity (as well as supplement existing data on mental health and substance use) during the pandemic – thus, this project would fill a gap in our knowledge.

3. Applicants

This seems like the ideal team to carry out the proposed project.

4. Budget

Staffing in first year seems high, specifically why 4 graduate students (at 50% time) plus a post-doc is needed. But overall, the budget seems reasonable given the scope of the project.

B. Comments on budget; formal recommendation

See previous comment. While some items on the budget seem high, others seem low (e.g., compensation for participants), thus I do not recommend a cut.

C. Integration of Sex and/or Gender in the Research Proposal

Gender not sex is integrated in the proposal. This is a strength of the application.

D. Issues to be flagged.

None
